# Incidence and determinants of diabetes-related lower limb amputations in Ghana, 2010–2015- a retrospective cohort study

**DOI:** 10.1186/s12902-019-0353-8

**Published:** 2019-03-01

**Authors:** Osei Sarfo-Kantanka, Fred Stephen Sarfo, Ishmael Kyei, Charles Agyemang, Jean Claude Mbanya

**Affiliations:** 1 Komfo Anokye Teaching Hospital, Endocrine and Diabetes Unit, P.O Box 1934, Kumasi, Ghana; 2 Department of Medicine, Komfo Anokye Teaching Hospital/ School of Medical Sciences, Kwame Nkrumah University of Science and Technology, Kumasi, Ghana; 3 Department of Public Health, Academic Medical Center, University of Amsterdam, Amsterdam, Netherlands; 4 Faculty of Medicine and Department of Biomedical Sciences, University of Yaoundé 1, Yaoundé, Cameroon

**Keywords:** Diabetes, Lower limb amputation, Ghana

## Abstract

**Background:**

Diabetes-related lower limb amputations (LLA) are associated with significant morbidity and mortality. Although the incidence has decreased over the past two decades in most High-Income Countries, the situation in Low-Middle Income Countries (LMIC), especially those in sub-Saharan Africa (SSA) is not clear. We have determined the incidence and determinants of diabetes-related LLA in Ghana.

**Methods:**

This was a tertiary-care-based retrospective cohort study involving patients enrolled in the diabetes clinic of Komfo Anokye Teaching Hospital, Ghana from 1st January 2010 to 31st December 2015 after a median follow-up of 4.2 years. Demographic characteristics and clinical variables at baseline were recorded. The primary outcome was new diabetes-related LLA in each year under study. Cox proportional hazard regression models were used to describe the associations of diabetes-related LLA.

**Results:**

The mean age at enrolment for the cohort was 55.9 ± 14.6 years, with a female preponderance (62.1%). The average incidence rate of diabetes-related LLA was 2.4 (95% CI:1.84–5.61) per 1000 follow-up years: increasing from 0.6% (95% CI:0.21–2.21) per 1000 follow up years in 2010 to 10.9% (95% CI:6.22–12.44) per 1000 follow-up years in 2015. Diabetes-related LLA was associated with increased age at enrollment (for every 10 year increase in age: HR: 1.11, CI: 1.06–1.22, *p* < 0.001), male gender (HR: 3.50, CI:2.88–5.23, *p* < 0.01), type 2 diabetes (HR 3.21, CI: 2.58–10.6, *p* < 0.001), high Body Mass Index (HR: 3.2, CI: 2.51–7.25 *p* < 0.001), poor glycemic control (for a percent increase in HbA1c, HR:1.11, CI:1.05–1.25, *p* = 0.03), hypertension (HR:1.14, CI:1.12–3.21 *p* < 0.001), peripheral sensory neuropathy (HR:6.56 CI:6.21–8.52 *p* < 0.001) and peripheral vascular disease (HR: 7.73 CI: 4.39–9.53, *p* < 0.001).

**Conclusion:**

The study confirms a high incidence of diabetes related-LLA in Ghana. Interventions aimed at addressing systemic and patient-level barriers to good vascular risk factor control and proper foot care for diabetics should be introduced in LMICs to stem the tide of the increasing incidence of LLA.

## Background

Diabetes complications continue to increase parallel to the exponential increase in the incidence of the disease worldwide [1, 2]. The greatest burden of these complications can be found in Low-Middle Income Countries (LMIC) of the world [3, 4]. Disorders of the foot represent one of the most prevalent and feared of the complications of diabetes [5]. It portends a high risk of lower limb amputation (LLA) and mortality [6, 7]. Evidence from resource-rich industrialised world shows a decrease in the incidence of diabetes-related LLA by 48–78% after the introduction of multidisciplinary foot care clinics [8–10], specified referral pathways and stringent diabetes foot education [11–13].

Diabetes care in LMICs especially those in sub-Saharan Africa (SSA) is beset with inherent organisational deficits including fragmentation of care, inadequate allocation of resources and unwavering attention to achieving glycemic targets [14, 15]. These factors have contributed to an increasing burden of complications prominent among them foot disorders [16, 17]. Although a high burden of non-traumatic LLA has recently been reported in Ghana [18], the role of diabetes on this burden is yet to be clarified.

The trajectories and determinants of diabetes-related LLA in LMICs are needed to provide a basis for comparison with incidence from other areas of high-quality diabetes care. Additionally, baseline data is required for the design and testing of locally appropriate foot care interventions if LLA from diabetes is to be reduced in LMICs.

This study aimed to determine the incidence of diabetes-related LLA in a cohort of patients enrolled in an outpatient tertiary clinic from 2010 to 2015. Secondly, we have identified the clinical factors that predict diabetes-related LLA in the cohort.

## Methods

### Profile of study area and population

We undertook a retrospective cohort study of patients who enrolled in the diabetes clinic of Komfo Anokye Teaching Hospital, a tertiary hospital in Kumasi, Ghana from 1st January 2010 to 31st December 2015. The hospital is situated in the central belt of Ghana and serves an estimated 10 million people from six out of the 10 regions of Ghana as well as other neighbouring countries. The diabetes clinic was established in 1992 and runs daily during the working week. Over 20,000 patients have enrolled in the clinic for follow up with the current active population estimated at 12,000 patients. The weekly attendance to the clinic range between 300 and 450 patients. The clinic is run by a team of Physicians/Diabetologist, nurses and dieticians. The study was approved by the Committee on Human Research Publication and Ethics of the School of Medical Sciences, Kwame Nkrumah University of Science and Technology, and the Komfo Anokye Teaching Hospital, Kumasi. We anonymised patient’s records/information before analysis.

### Data collection

We trained research assistants for 2 days on how to retrieve and extract relevant data from medical records of patients and return the files back to their original location. The lead author examined approximately 10 % of all data recording sheets for competence and consistency. We retrieved folders of patients who enrolled in the clinic from 1st January 2010 to 31st December 2015 by satisfying the criteria of the World Health Organisation for the diagnosis of diabetes i.e. an elevated fasting plasma glucose level (≥ 7 mmol/L) on two occasions, or oral glucose tolerance test ≥11.1 mmol/l [19].

Data extracted from patient folders included demographic and relevant clinical information at enrolment; age, gender, age at diagnosis, alcohol intake and smoking status. Others included weight (kg), body mass index (BMI), blood pressure, glycated haemoglobin (HbA1c), lipid profile and estimated Glomerular Filtration Rate (eGFR). Neuropathic and vascular symptoms including paresthesia, intermittent claudication were recorded. Peripheral neuropathy(PN) was diagnosed using a combination of symptoms such as paresthesia’s, numbness as well as monofilament and vibration sense testing. Peripheral artery disease (PAD) was diagnosed based on symptoms such as intermittent claudication allied to ankle-brachial index measurements (less than 0.9), Doppler ultrasonography and magnetic resonance imaging angiography. Patients who had undergone diabetes-related LLA including when, type and the limbs affected were identified from the folders and recorded. This information was crosschecked from the theatre records of the hospital in most cases KATH where the surgeries were done. Any LLA performed distal to the ankle joint was classified as minor, whiles any LLA performed through or proximal to the ankle joint was classified as major. [20] We excluded patients who had a diabetic foot ulcer at enrolment, as well as those with diabetes-related LLA before enrolment to focus on only patients who developed foot ulcers and LLA after enrolment into the diabetes clinic. We also excluded patients who were lost to follow up or those who died before completing one full year of follow up after enrolling in the clinic.

### Statistical analysis

We described data using means and medians and compared them using either the Student t-test or Mann Whitney U-test for paired comparisons depending on whether the variables were parametric or non-parametric. We tested differences in frequencies for significance using the Chi-square test or Fishers Exact tests. We calculated incidence rates as the number of patients with new LLA per the total study group person-years. We created Cox proportional hazard regression analysis models to determine factors that predicted LLA in the cohort. Variables included in the model were: age (years) at presentation, gender, type of diabetes, lipid status, renal function, and symptoms of peripheral neuropathy. Statistical significance was set at a *p*-value of < 0.05 (two-sided). Statistical analysis was performed using Graph Pad Prism 7 and SPSS ver. 20.

## Results

### Characteristics

A total of 3722 patients enrolled in the diabetes clinic between 1st January 2010 and 31st December 2015. We excluded 127 of them because they presented to the clinic with foot disorders and 325 because they had been lost to follow-up before a year of enrolment had passed. A total of 3143 patients were therefore included in the analysis. The clinical characteristics of the patients included in the study are shown in Table 1. The median duration of follow up was 4.2 years. The mean age at diagnosis was significantly lower among those with diabetes-related LLA. The mean duration of diabetes before referral to the diabetes clinic was significantly higher for those who underwent LLA. Almost all diabetes-related LLA occurred in type 2 diabetes patients (97.8%). At baseline, those with LLA had significantly higher mean systolic and diastolic blood pressures, a higher proportion of dyslipidaemia, poorer glycemic controls and obesity (all *p* < 0.001). Patients with LLA had lower rates of statins, antiplatelet therapy, ACE inhibitors and Angiotensin Receptor Blockers, metformin and insulin prescriptions at baseline compared to those without diabetes-related LLA (*p* < 0.001). Symptoms of PSN and PVD were reported frequently in those with LLA at baseline compared to those without LLA (all *p* < 0.001). There was no significant difference between those with LLA and those without LLA regarding alcohol use and cigarette smoking at baseline.

**Table 1:** Baseline characteristics of patients by presence of Lower Limb Amputation in diabetes clinic, 2010–2015

Parameters	Combined	Lower Limb Amputation	No Lower Limb Amputation	*p*-value
	(*n* = 3143)	(*n* = 78)	(*n* = 3065)	
Gender				
Female, n (%)	1952 (62.1)	30 (38.5)	1922(62.7)	***< 0.001***
Age, mean ± SD	59.5 ± 14.6	62.2 ± 11.8	58.5 ± 14.9	***0.02***
Age at diagnosis, mean ± SD	49.2 ± 11.2	46.2 ± 10.2	52.2 ± 11.6	***< 0.001***
Duration of diabetes	10.2 ± 5.6	10.9 ± 6.8	9.6 ± 4.9	0.08
Location of domicile				0.29
Urban	2567 (81.7)	52 (56.7)	2515 (82.1)	***0.002***
Semi-urban	409 (13.0)	8 (10.3)	401 (13.1)	0.61
Rural	167 (5.3)	18 (23.1)	149 (4.7)	***< 0.001***
Highest educational attainment				***0.03***
None	454 (14.3)	23 (29.5)	431 (14.1)	***< 0.001***
Primary	604 (19.2)	23 (29.5)	601 (19.6)	***0.04***
Secondary	1389 (44.2)	26 (33.3)	1644 (53.6)	***< 0.001***
Tertiary	695 (22.1)	6 (7.8)	389 (12.7)	0.23
Employment status				
Skilled academic	298 (9.5)	21 (26.9)	277 (9.0)	***< 0.001***
Manual worker	1763 (56.1)	32 (41.0)	1731 (56.4)	***0.01***
Retired	426 (13.6)	16 (20.5)	410 (13.4)	0.09
Unemployed	517 (16.4)	9 (11.5)	508 (16.8)	0.28
Duration before referral to Centre, mean ± SD (years)	3.8 ± 2.5	5.4 ± 1.7	1.3 ± 2.2	***0.001***
Diabetes type				0.25
Type 1	192 (6.1)	2 (2.6)	156 (5.1)	0.43
Type 2	2793 (88.9)	76 (97.8)	2717 (88.6)	***0.001***
Others	168 (5.3)	0 (0)	192 (6.3)	–
Vascular risk factors				
Hypertension, n (%)	2678 (85.2)	74 (94.9)	2604 (84.6)	0.02
Dyslipidemia, n (%)	1897 (60.4)	59 (75.6)	1838 (60.0)	0.004
Cigarette smoking n (%)	36 (1.1)	9 (11.5)	27 (0.9)	0.84
Alcohol Use, n (%)	756 (24.1)	18 (23.1)	738 (24.1)	0.43
Waist-to-Hip ratio, mean ± SD	0.93 ± 0.09	0.95 ± 0.05	0.94 ± 0.08	0.18
Body Mass Index, mean ± SD	28.0 ± 3.3	26.0 ± 3.7	27.1 ± 3.6	***0.007***
Systolic blood pressure, mmHg	143 ± 35.1	156 ± 43.1	130 ± 18.9	***0.0001***
Diastolic blood pressure, mmHg	89.3 ± 23.2	96.2 ± 22.1	82.4 ± 14.4	***0.0001***
Therapy				
Lipid-lowering therapy, n (%)	1245 (39.6)	12 (15.4)	1233 (40.2)	***< 0.0001***
Antiplatelet therapy, n (%)	1345 (42.8)	23 (29.5)	1322 (43.1)	***0.01***
ACE/ARB Inhibition, n (%)	2567 (81.7)	35 (44.9)	2532 (80.6)	***< 0.0001***
Calcium channel blockers, n (%)	2064 (65.7)	64 (82.1)	2000 (65.3)	***< 0.0001***
Centrally acting agents, n (%)	478 (15.2)	13 (16.7)	465 (15.1)	0.75
Diuretics, n (%)	1567 (49.9)	12 (15.4)	1355 (44.2)	***< 0.001***
Metformin, n (%)	2975 (94.7)	48 (61.5)	2927 (94.5)	***< 0.001***
Sulphonylureas, n (%)	2456 (78.1)	63 (80.8)	2393 (78.1)	0.68
DPP4 Inhibitors, n (%)	102 (3.2)	1 (1.3)	101 (3.3)	0.52
Insulin, n (%)	624 (19.9)	2 (2.6)	622 (20.3)	***< 0.001***
Glycemic control				
Fasting blood glucose (mmol/L)	8.9 ± 5.2	12.6 ± 5.6	7.9 ± 4.5	***< 0.0001***
HbA1c (%)	8.1 ± 2.1	10.4 ± 3.2	7.5 ± 4.2	***< 0.0001***
Estimated Glomerular Filtration Rate	42.2 ± 9.8	29.2 ± 6.8	66.4 ± 5.6	***< 0.0001***
Peripheral sensory neuropathy	867 (27.6)	66 (84.6)	801 (26.1)	***< 0.0001***
Peripheral Vascular Disease	87 (2.8)	26 (33.3)	61 (2.0)	***< 0.0001***

*n number, SD standard deviation, ACE angiotensin converting enzyme, ARB angiotensin receptor blocker, DPP4, dipeptidyl peptidase inhibitor, HbA1c glycated hemoglobin*

### Incidence of amputations

**Fig. 1:**
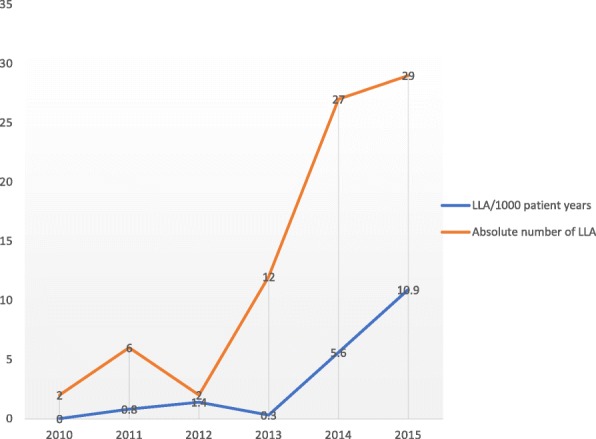
Diabetes-related lower limb amputation in Central Ghana

The absolute number of diabetes-related LLA among our cohort increased from 2 in 2010 to 29 in 2015. The average incidence rate of diabetes related-LLA was 2.4 (95% CI:1.84–5.61) per 1000 follow up years: increasing from 0.6 (95% CI:0.21–2.21] per 1000 follow up years in 2010 to 10.9 (95% CI:6.22–12.44) per 1000 follow up years in 2015 (Fig. 1).

### Causes of amputations

**Fig. 2:**
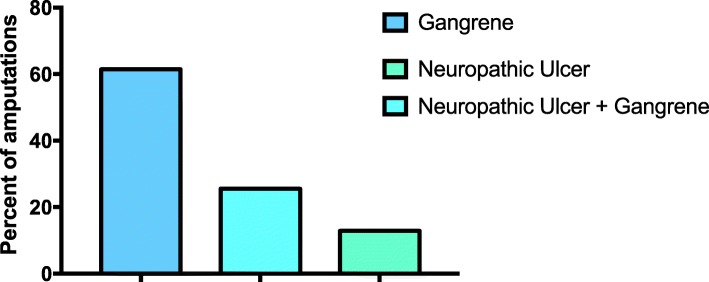
Causes of diabetes-related lower limb amputations in central Ghana

The main causes of amputation included PVD, i.e. 63% of LLA, PSN; 24 and 13% were due to a combination of the two (Fig. 2).

### Types of amputations

67.9% of LLA was classified as major LLA, 32.1% were minor. 42.3% of the LLA were above the knee, 28.2% were below the knee, 5.1% had bilateral above-knee amputations, and 5.1% had bilateral below knee amputations. Of the 25 minor amputations: 15.3% had a right digital amputation, 10.3% had left digital amputation, and 6.4% had bilateral digital amputations (Table 2).

**Table 2:** Type of Amputations among study participants

Type of Amputation	Total Number (*n* = 78)	Males (*n* = 62)	Females (*n* = 16)
Major Amputations (*n* = 53)			
Right Above Knee Amputation	25 (32.0)	20 (25.6)	5 (6.4)
Right below Knee Amputation	13 (16.7)	10 (12.8)	3 (3.8)
Left Above Knee Amputation	4 (5.1)	3 (3.8)	1 (1.3)
Left Below Knee Amputation	4 (5.1)	4 (6.5)	0 (0)
Bilateral Above Knee Amputation	4 (5.1)	3 (3.8)	1 (1.3)
Bilateral below Knee Amputation	3 (3.8)	3 (3.8)	0 (0)
Minor Amputations (*n* = 25)			
Right Digital Amputation	12 (15.3)	10 (12.8)	2 (2.6)
Left digital amputation	8 (10.3)	5 (6.4)	3 (3.8)
Bilateral Digital Amputation	5 (6.4)	4 (6.5)	1 (1.3)

### Predictors of lower limb amputations

Independent factors associated with LLA in our diabetes cohort are shown in Table 3: age at presentation; every 10-year increase in age increased the risk of LLA by 11%, male gender was associated with 3.5 times increased the risk of LLA. Patients with type 2 diabetes had eight times increased the risk of LLA compared to those with other forms of diabetes. Every 5 kg/m^2^ increase in BMI increased the risk of LLA three times. A percentage increase in HbA1c increased the risk of LLA by 11%. The presence of hypertension increased the risk of LLA by 14%. The presence of PSN and PVD increased the risk of LLA by about 7 and 8-fold respectively.

**Table 3:** Predictors of Lower extremity amputations

Predictor	Unadjusted HR (95% CI)	*P*-Value	Adjusted HR (95% CI)	*P*-Value
Gender				
Male	5.28 (3.11–8.96)	< 0.001	3.50 (2.88–5.23)	**< 0.001**
Female	1			
Age at presentation				
10 years’ age increase	1.40 (1.19–1.65)	< 0.001	1.11 (1.06–1.22)	**< 0.001**
Duration of diabetes				
5 year increase	2.40 (0.92–3.14)	< 0.07	–	
Type of DM				
Type 1	1		1	
Type 2	6.20 (3.13–9.43)	< 0.001	8.21 (2.58–10.7)	**< 0.001**
Specific type	0.50 (0.32–1.21)	0.32		
Body mass index				
Each 5 kg/m^2^ increase	5.3 (3.11–8.96)	< 0.001	3.2 (2.51–7.25)	**< 0.001**
Glycemic control				
A percentage increase in HbA1c	1.23 (1.08–1.42)	0.003	1.11 (1.05–1.25)	0.03
Dyslipidaemia				
Present	0.84 (0.51–1.36)	0.47	–	–
Absent	1			
Complications				
Hypertension	1.51 (1.02–1.71)	< 0.001	1.14 (1.12–3.21)	**< 0.001**
Nephropathy	1.6 (0.8–2.3)	0.07	–	**–**
A 10/mL/min/1.73 m ^2^ drop in eGFR				
Peripheral sensory neuropathy	7.28 (6.38–9.21)	< 0.001	6.56 (6.21–8.52)	**< 0.001**
Peripheral Vascular Disease	9.76 (5.67–12.21)	< 0.001	7.73 (4.39–9.53)	**< 0.001**

## Discussion

In this retrospective cohort study spanning a period of 6 years, the incidence of diabetes-related LLA within a diabetes cohort attending a tertiary referral centre in Ghana rose from 0.6 per 1000 follow up years in 2010 to 10.9 per 1000 follow up years in 2015. We identified gangrene/PVD as the leading cause of diabetes-related LLA in the cohort. Additionally, diabetes-related LLA in our cohort was associated with uncontrolled vascular risk factors and poor glycemic control at baseline. Our study thus confirms a high burden of foot disorders and diabetes-related LLA among diabetes patients in LMICs [16–18]. This finding is in contrast with those from industrialised countries where the incidence of diabetes-related LLA has reduced over the past two decades [21–23]. In studies in England, the United States and Australia, the incidence of diabetes-related LLA reduced significantly with rates ranging from 5.5 to 36 per 100,000 people in the general population over a decade [24, 25]. The reduced rate of diabetes-related LLA in industrialised countries compared to those recorded in our study and studies from other LMICs can be attributed to the introduction of multidisciplinary diabetes foot care clinics, streamlined care pathways, and patient education on foot screening [8–13]. Although the study was sited at a tertiary referral hospital where mostly the patients referred, have serious forms of diabetes often with complications, the increasing trend of LLA is alarming. However in Ghana, as in most other LMICs where a rising trend of diabetes-related LLA has been observed, measures such as lack of resources, high patient load, absence of suitable facilities and trained personnel have been identified as major contributors [26]. Additionally, in Ghana, the life expectancy of the population including those with diabetes has increased over the years as a result of improvement in the economic conditions of the country with additional implications for medication purchasing abilities as well as improvement in dietary choices [27].

The commonest predictor of diabetes-related LLA in our cohort as revealed by this study was PVD, like findings from other studies in Europe [28, 29]. A general trend has been observed among people with diabetes in LMICs with a shift towards an increase in cardiovascular disease and its risk factors. This was observed in our study, with a high prevalence of uncontrolled vascular risk factors such as hypertension and hyperglycemia. However, only 15.4, 29.5 and 44.9% of those with diabetes-related LLA were on lipid-lowering, antiplatelet and renin-angiotensin-aldosterone inhibitor therapy at baseline. This therapeutic inertia in the face of overwhelming benefit of these medications collaborates findings that show an association between diabetes-related LLA rates and poor vascular risk factor control among diabetes patients [30]. This situation is compounded by the absence of established vascular imaging services and surgeons in our setting and with other LMICs. Thus any attempt to reduce or treat these vascular complications after they become established is seriously hampered. It is not by coincidence therefore that most studies reporting a decrease in the incidence of diabetes-related LLA in patients with diabetes have been performed by researchers in highly specialised diabetic foot care units as well as those working in centres with well-established vascular services [31].

Our study shows type 2 diabetes patients were at eight times increased risk of diabetes-related LLA. Although type 1 diabetes patients were proportionally lower in numbers compared to type 2 diabetes patients, it is suggested that type 1 diabetes patients are more strictly controlled for glycaemia and other risk factors for diabetic foot disease; in contrast, many patients with type 2 diabetes have less aggressive management of these risk factors as well as an abundance of vascular risk factors usually mediated by insulin resistance [32].

The increasing incidence of diabetes-related LLA brings into sharp focus the care of diabetes patients in Ghana and other LMICs. Since diabetes foot and diabetes-related LLA often serve as an indicator of diabetes management, severity and quality of diabetes care. It may be concluded that diabetes care in the middle belt of Ghana is not optimum. Diabetes care in Ghana as in most LMIC focus attention almost entirely on achieving glycemic targets with a limited effort at improving patient education, self-care practices and control of vascular risk factors such as hypertension and obesity. Patients are therefore left bereft of knowledge, delay in seeking treatment and lack of proper foot care [26].

This study has limitations worth noting. It was conducted in a single centre among a cohort of diabetes patients thus cannot be generalised to the entire Ghanaian diabetes population and indeed LMICs. Additionally, a significant risk of bias is inherent due to the retrospective design and the fact that the study was based on patients from a single centre. This, however, is the first study to the best of our knowledge to provide a detailed assessment of a longitudinal record of LLA among diabetes patients as well as the predictors of LLA in SSA. This study has raised several important questions which are pertinent if we are to reduce the incidence of LLA in resource-limited settings such as ours where health professionals are in short supply, and a severe lack of investigative tools exists. In this environment, effective prevention programs that are feasible, acceptable, timely and sustainable are urgently needed amid a rising epidemic of diabetes and its complications. Thus community-based studies are required to give a clearer picture of the extent of diabetic-related LLA in LMIC and interventions urgently crafted to address them.

## Conclusion

In conclusion, the present study has shown an increased incidence of diabetes-related LLA in a cohort of patients with diabetes referred to a tertiary referral centre in Ghana. This increase in the rate of LLA was associated with a high prevalence of vascular risk factors. This call for better management of diabetes and vascular risk factors as well as initiatives to raise awareness and access to services focused on diabetes-related foot health. Also, the availability of advice on other preventive podiatric measures (such as appropriate footwear and strategies to offload pressure points) and regular vascular assessments and other intervention should be increased.

## Abbreviations

BMI: Body Mass Index
HbA1c: Glycated haemoglobin
LLA: Lower Limb Amputations
LMIC: Low middle-income countries
SSA: Sub-Saharan Africa
